# Rheumatoid Arthritis from Pathogenesis to Therapeutic Strategies

**DOI:** 10.3390/jcm9082562

**Published:** 2020-08-07

**Authors:** Ruediger B. Mueller, Paul Hasler

**Affiliations:** Division of Rheumatology, Medical University Department, Kantonsspital Aarau, 5001 Aarau, Switzerland

Rheumatoid arthritis (RA) is a chronic inflammatory disease that leads to joint destruction. Various therapeutic agents have been showed to halt disease progression in clinical studies. In this special issue, we cover subjects from the periodontal condition of RA patients [1,2] to therapeutic strategies [3], and patient related outcomes [4,5], accompanied by the most extensive review ever on methotrexate (MTX) use in RA [6].

Eriksson et al. [1] describe that the subgingival plaque of RA patients with moderate/severe RA was enriched with abundant bacteria of different bacterial strains typical for periodontitis. Interestingly, ACPA also positivity correlated with moderate to severe periodontitis. Rinaudo-Gaujous et al. [2] showed that MMP-3 (matrix metalloproteinase 3), a marker of periodontal disease and bone and cartilage degradation, decreases subsequent to newly introduced infliximab therapy together with a reduction of disease activity. The interesting question is whether periodontitis primarily improves due to the reduction of disease activity, or whether improved arthritis leads to less pain during dental brushing and improved dental care remains, so far, unsolved.

Köhler et al. [7] reviewed the available methods for treatment of RA, while Mueller et al. [3] discussed how combination of the whole therapeutic armamentarium (new onset biologic agent, intra-articular and oral glucocorticoids, and optimization of conventional synthetic DMARDs) leads to a vastly improved outcome in a randomized clinical study. ACR 20, 50, and 70 response rates were achieved in 90.5%, 76.2%, 71.4%, an outcome that has so far not been achieved in a clinical trial of RA. The same group also reports the most extensive real-life experience of RA patients treated with tofacitinib in this issue [8]

Taylor et al. [6] wrote the largest and most comprehensive review on MTX, covering the pharmacology, the flexibility and efficacy and cost/benefit of the drug. Included among many other topics are the potential toxicities of MTX. 

Hirter et al. [9] reviewed the literature on pseudo-erosions and came to three conclusions: (A) Pseudo-erosions may be related to normal anatomy or technical artefacts. (B) So-called calcified zones can be part of classical anatomical structures, such as subchondral, sub-tendinous or -ligamentous bone. (C) As a caveat, a real arthritic erosion can develop at the site of a pseudo-erosion. 

In two post-hoc analyses of the RA BEAM Study Fautrel et al. [4] and Taylor et al. [5] demonstrated that in RA patients with moderately to severely active RA despite MTX treatment, the addition of baricitinib may be more effective in improving pain and physical function than placebo or addition of adalimumab.

In summary, in this special issue the disease of RA and its therapy is described from different angles to provide a broad and profound insight into the disease.
